# Dynapenic Abdominal Obesity and Cognitive Impairment in Type 2 Diabetic Patients: A Single‐Center Cross‐Sectional Study

**DOI:** 10.1155/ije/6060666

**Published:** 2026-03-12

**Authors:** Yinghao Li, Yan Pi, Xin Ye, Mei Mei, Kun Liao, Fengning Chuan, Rong Li, Bo Zhou

**Affiliations:** ^1^ Department of Endocrinology, The First Affiliated Hospital of Chongqing Medical University, Chongqing, 400016, China, cqmu.edu.cn; ^2^ Department of Endocrinology, The Second Affiliated Hospital of Army Medical University, Chongqing, 400037, China

**Keywords:** cognitive impairment, dynapenic abdominal obesity, grip strength, Type 2 diabetes

## Abstract

**Background:**

The relationship between obesity and cognitive dysfunction remains controversial. Dynapenic abdominal obesity (DAO) is characterized by a combination of abdominal obesity and low muscle strength. However, as a unique subtype of obesity, the relationship between DAO and cognitive function in patients with Type 2 diabetes (T2D) is not clear.

**Materials and Methods:**

A total of 270 middle‐aged and older T2D patients were included in the study and divided into four groups: dynapenic obesity (DO), nondynapenic obesity (NO), dynapenic nonobesity (DN), and nondynapenic nonobesity (NN). Patient demographics, lifestyle habits, metabolic and inflammatory markers, diabetes‐related characteristics, grip strength (GS), anthropometry, and Montreal Cognitive Assessment (MoCA) scores were recorded. Multiple linear regression was used to analyze the relationships among GS, waist circumference (WC), DAO, and MoCA scores, and multiple logistic regression was used to identify the factors influencing DAO.

**Results:**

Compared with NN patients, patients with DO tended to be older and female. These patients had higher insulin resistance indices and inflammatory indicators, lower hemoglobin levels and estimated glomerular filtration rate (eGFR), and a greater burden of diabetes. In addition, the DO group presented lower gait speeds and greater body fat percentage and limb fat mass. More importantly, they had lower MoCA scores. Notably, DAO and lower GS were associated with lower MoCA scores. The following factors influence DAO: age, gait speed, and diabetic retinopathy.

**Conclusions:**

Among middle‐aged and older individuals with T2D, DAO is independently associated with cognitive impairment and presents phenotypic features linked to age and diabetes.

## 1. Introduction

The prevalence of Type 2 diabetes (T2D) is continuously increasing as the global population ages, lifestyles change, and social pressures increase [1]. According to data from the International Diabetes Federation in 2021, T2D affects approximately 536.6 million people globally, with a staggering 20% prevalence rate among elderly individuals over 60 years of age [2]. Compared with their young counterparts, middle‐aged and older individuals with T2D are more susceptible to polypharmacy, malnutrition, and age‐related comorbidities such as cardiovascular diseases, sarcopenic obesity, frailty, and cognitive disorders, in addition to suffering from traditional complications [3]. A recent meta‐analysis revealed that, compared with nondiabetic individuals, the risk of cognitive impairment is markedly increased by 25%–91% in individuals with T2D [4]. Even worse, once diabetic patients have cognitive dysfunction, they often exhibit a diminished capacity for self‐management, which can result in increased reliance on healthcare for diabetes symptoms and lead to a decrease in treatment compliance, acute severe hypo‐ or hyperglycemic episodes, and worsening of complications. All these factors, in turn, exacerbate cognitive impairment, perpetuating a vicious cycle [5]. Therefore, early identification of cognitive decline and timely intervention are crucial for diabetes care, especially in elderly individuals.

To date, data on the relationship between obesity and cognitive impairment are still conflicting. One meta‐analysis published in 2020 reported that midlife obesity, defined by body mass index (BMI), is a strong predictor of cognitive impairment in older individuals [6]. However, another observational cross‐sectional study revealed that being overweight in older adults is associated with a decreased likelihood of adverse cognitive outcomes [7]. Similarly, a large‐scale cohort study spanning 2 decades, involving two million people, also indicated that an increase in BMI during both middle age and old age is strongly associated with a reduced incidence of dementia [8]. The reasons for the inconsistencies mentioned above are not fully understood: the different study designs, sample populations surveyed, obesity measures, and other confounding factors may be involved to varying degrees. Among these factors, BMI‐defined obesity is particularly noteworthy because BMI is strongly affected by muscle mass changes. More importantly, previous studies have almost unanimously shown that sarcopenia could effectively predict the development of future cognitive decline [9]. In addition, available data have revealed that a reduction in muscle strength is more indicative of dementia than is a reduction in skeletal muscle mass in the general population [10].

Dynapenic abdominal obesity (DAO) is a novel subtype of obesity that was identified in recent years and is characterized by abdominal obesity coupled with a decline in muscle strength associated with aging or illness [11]. A number of studies have indicated that individuals with T2D tend to be more likely to experience a decline in muscle mass and strength but an increase in visceral fat [12]. Moreover, another study suggested that DAO patients are at increased risk of developing metabolic syndrome [13]. Furthermore, a cross‐sectional study published in 2022 demonstrated that DAO is significantly correlated with mild cognitive impairment (MCI) in patients with cardiometabolic disease [14]; however, very few studies have explored the relationship between DAO and cognition among the population with diabetes to date. Therefore, to determine the role of DAO as a surrogate marker for cognitive outcome, this study first investigated the phenotypic characteristics of patients with DAO among middle‐aged and older patients with T2D. The relationship between DAO and cognitive impairment was subsequently observed after adjusting for potential confounders. Finally, we analyzed whether one of the two components of DAO, namely, grip strength (GS) or waist circumference (WC), is independently associated with cognitive impairment.

## 2. Materials and Methods

### 2.1. Participants

**FIGURE 1:**
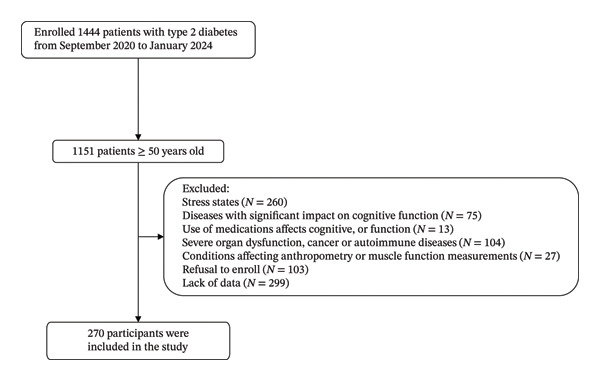
Flowchart of study subjects.

Participants were consecutively enrolled from the Department of Endocrinology, the First Affiliated Hospital of Chongqing Medical University, between September 2020 and January 2024. The inclusion criteria were as follows: (1) previously diagnosed with T2D based on the 1999 World Health Organization diagnostic criteria [15]; (2) aged ≥ 50 years; and (3) capable of self‐care and communication in daily life. The exclusion criteria were as follows: (1) acute complications of diabetes, serious infections, surgery, or other stress states; (2) diseases that significantly affect cognitive function, such as neurological disorders (Alzheimer’s disease, vascular dementia, brain tumors, etc.), vitamin B12 or folate deficiency, hypothyroidism, hypercalcemia, and so on; (3) the use of medications that affect cognitive function, such as antipsychotics, antidepressants, benzodiazepines, or anticholinergic drugs, and so on; (4) severe organ dysfunction, cancer, or autoimmune diseases; (5) conditions affecting anthropometry or muscle function measurements, such as edema, limb amputation, or injury, and so on; and (6) inability or unwillingness to cooperate with the study. Each participant completed questionnaires and scales, blood tests, and physical examinations. The flowchart of the subjects is shown in Figure 1, ultimately resulting in 270 participants enrolled in this study.

### 2.2. Anthropometry and Muscle Function

WC was measured at the midpoint from the lower rib to the iliac crest using a tape while the subject stood with a relaxed abdomen. Body composition indicators such as appendicular skeletal muscle mass (ASM), total body fat percentage (BF%), limb fat mass, and estimated visceral adipose tissue area (eVATA) were measured via dual‐energy X‐ray absorptiometry (DXA, Discovery A, S/N 87352, Hologic, Bedford, USA) by a uniformly trained technician. The ASM was determined by either taking the square of an individual’s height or their body weight and was subsequently expressed as the ASM index (ASMI) or the ratio of ASM to body weight (ASM/W), respectively. Both hands’ GSs were measured twice via an electronic spring dynamometer (EH101, CAMRY, Guangdong, China) while the subject stood with extended elbows and exerted maximum effort, and the highest value was recorded as the maximum GS. The time for a 6‐m walk at a normal pace was recorded twice, and the gait speed was determined from the trials’ average.

### 2.3. Group

According to the criteria of the Asian Working Group for Sarcopenia (AWGS) in 2020, dynapenia is defined as a GS < 28 kg for males and < 18 kg for females [16]. Following the definition of obesity by the Chinese Nutrition Society’s Obesity Prevention and Control Section in 2022, abdominal obesity is defined as a WC ≥ 90 cm for males and ≥ 80 cm for females [17]. Based on this conceptual framework, the subjects were divided into four groups: dynapenic obesity (DO), nondynapenic obesity (NO), dynapenic nonobesity (DN), and nondynapenic nonobesity (NN).

### 2.4. Assessment of Cognitive Function

The Montreal Cognitive Assessment (MoCA), with a total score of 30 points, was used to evaluate cognitive function. When the subject’s years of education were ≤ 12 years, an additional point was added to the scores. Based on Chinese expert consensus on the diagnosis of MCI 2021, MCI was defined as a MoCA score < 26 points [18]. Permission to use the MoCA has been granted by MoCA Cognition.

### 2.5. Data Collection

Demographic characteristics (age, sex, BMI, smoking history, drinking history, etc.) were obtained from the electronic medical records system. Additionally, biochemical indicators (fasting blood glucose, glycated hemoglobin [HbA1c], fasting C‐peptide, blood lipid profile, estimated glomerular filtration rate [eGFR], hemoglobin, 25‐hydroxyvitamin D [25(OH)D], high‐sensitivity C‐reactive protein [hsCRP], etc.), diabetes‐related features (duration, complications, and medications for diabetes), antihypertensive drugs, statins, and comorbidities (hypertension, coronary heart disease [CHD], stroke, etc.) were also recorded.

The homeostasis‐model assessment for insulin resistance (C‐peptide) (HOMA‐IR [CP]) formula was used to calculate the index of insulin resistance [19]. The Diabetes Complications Severity Index (DCSI) was used to assess the severity of diabetes burden [20].

### 2.6. Other Questionnaires and Scales

Other demographic characteristics (years of education, marital status, living alone, income level, occupation, types of diet) and hypoglycemic events were obtained through standardized questionnaires by uniformly trained researchers. Monthly income ≤ 2000 RMB, < 6000 RMB, and ≥ 6000 RMB were defined as low, middle, and high income, respectively. Nine types of diets (meat, vegetables, fruits, eggs, milk or dairy products, beans or bean products, fish or seafood, nuts, and tea) were collected, and 1 point was recorded for each category of the above foods consumed at least once a week. Hypoglycemic events were defined as the occurrence of autonomic symptoms of hypoglycemia (palpitations, tremors, sweating, hunger, etc.), neuroglycopenic symptoms (coma, confusion, etc.), or blood glucose levels ≤ 3.9 mmol/L within the last year [21].

The International Physical Activity Questionnaire‐Short Form (IPAQ‐SF) was employed to evaluate the intensity of physical activity among participants, categorized into low, moderate, and high levels [22]. The Patient Health Questionnaire‐9 (PHQ‐9) was used to measure depression, with scores ≥ 5 indicating mild depression, scores ≥ 10 indicating moderate‐severe depression, and scores ≥ 20 indicating severe depression [23]. The Pittsburgh Sleep Quality Index (PSQI) was used to assess sleep quality, with scores ≤ 5 indicating fair sleep quality, scores > 5 indicating good sleep quality, and scores > 15 suggesting poor sleep quality [24]. All the assessments were performed by uniformly trained researchers.

### 2.7. Statistical Analysis

The normality of the data was assessed via the Shapiro‒Wilk test. Continuous variables are expressed as the means (standard deviations, SDs) or medians (interquartile ranges, IQRs), whereas categorical variables are presented as numbers (%). One‐way ANOVA or the Kruskal‒Wallis test was used to compare overall differences in continuous variables among the four groups, and post hoc comparisons were made via the LSD test or the Mann‒Whitney *U* test to assess differences between groups. The chi‐square test or Fisher’s exact test was used to compare differences in categorical variables among and between groups. *p* values were adjusted via the Bonferroni correction, and a two‐sided *p* value < 0.05 was considered statistically significant. The association between DAO and MoCA scores was evaluated via multiple linear regression. Three models were designed: Model 1 was unadjusted; Model 2 was adjusted for age, sex, smoking status, alcohol intake, education level, income, living alone, and being depressed; and Model 3 was further adjusted for DCSI and HbA1c. Additionally, multiple linear regression was conducted to explore the relationship between GS or WC and MoCA scores. Finally, to explore the influencing factors of DAO, we treated DAO as a binary dependent variable and considered demographics, lifestyle habits, nutritional and inflammatory markers, and diabetes‐related characteristics as independent variables, performing univariate logistic regression for each. Ultimately, the independent variables with *p* < 0.1 in the univariate logistic regression were included in the multivariate logistic regression, and the receiver operating characteristic (ROC) curves were plotted. All analyses were conducted via IBM SPSS Statistics Version 26.0.

## 3. Results

### 3.1. Characteristics of Participants

The clinical profiles of the participants, categorized by dynapenia status and abdominal obesity status, are displayed in Table 1 and Supporting Table A. A total of 115 females and 155 males were included in the study, with an average age of 66.0 (59.0, 71.0) years. In total, 56 (20.7%), 121 (44.8%), 32 (11.6%), and 61 (22.6%) subjects were in the DO, NO, DN, and NN groups, respectively. Compared with the NN controls, the patients with DO tended to be older and were more likely to be female, although there were no significant differences in smoking history, alcohol consumption, education level, marital status, living alone, income, occupation, diet variety, or physical activity level. Similarly, patients with NO were more likely to be female, whereas patients with DN had a more limited diet variety than patients with NN. Compared with the NN group, the DO group had higher levels of fasting C‐peptide, HOMA‐IR, and hsCRP, with lower levels of hemoglobin and eGFR. There was no significant difference in fasting blood glucose, HbA1c, the blood lipid profile, albumin, or 25(OH)D levels between these two groups. Additionally, the NO group had higher fasting C‐peptide and hsCRP levels than the NN group did, and the DN group had lower eGFRs and hemoglobin levels than the NN group did.

**TABLE 1:** The demographics and biochemical indicators of participants.

	**Category**	**Overall**	**Dynapenic obesity**	**Nondynapenic obesity**	**Dynapenic nonobesity**	**Nondynapenic nonobesity**	***p***
***n* (%)**		**270 (100.0)**	**56 (20.7)**	**121 (44.8)**	**32 (11.6)**	**61 (22.6)**	

*Demographics*							
Age (years)		66.0 [59.0, 71.0]	70.5 [64.0, 74.3]^b^ ^,^ ^d^	65.0 [58.0, 71.0]^a^	66.5 [61.8, 73.3]	63.0 [59.0, 67.0]^a^	< 0.001
Female (%)		115 (42.6)	33 (58.9)^d^	54 (44.6)^d^	14 (43.8)	14 (23.0)^a^ ^,^ ^b^	0.001
Smoke (%)		94 (34.8)	17 (30.4)	40 (33.1)	10 (31.2)	27 (44.3)	0.357
Drink (%)		82 (30.4)	13 (23.2)	40 (33.1)	7 (21.9)	22 (36.1)	0.284
Education (years) (%)	< 7	51 (18.9)	20 (35.7)^b^	13 (10.7)^a^ ^,^ ^c^	9 (28.1)^b^	9 (14.8)	0.001
	7–12	141 (52.2)	25 (44.6)	65 (53.7)	18 (56.2)	33 (54.1)	
	> 12	78 (28.9)	11 (19.6)	43 (35.5)	5 (15.6)	19 (31.1)	
Diet variety scores		7.00 [6.00, 8.00]	7.00 [5.75, 8.00]	8.00 [7.00, 8.00]^c^	6.00 [5.00, 7.00]^b^ ^,^ ^d^	7.00 [6.00, 8.00]^c^	< 0.001
Physical activity (%)	Low	43 (16.0)	5 (8.9)	23 (19.0)	5 (15.6)	10 (16.7)	0.703
	Medium	171 (63.6)	43 (76.8)	75 (62.0)	19 (59.4)	34 (56.7)	
	High	55 (20.4)	8 (14.3)	23 (19.0)	8 (25.0)	16 (26.7)	

*Metabolic indicators*							
Fasting blood‐glucose (mmol/L)		7.90 [6.40, 10.00]	8.60 [6.80, 11.80]	7.70 [6.00, 9.30]	8.50 [6.20, 12.50]	7.70 [6.57, 9.10]	0.185
HbA1c (mmol/mol)		73.00 [54.00,96.00]	68.00 [55.00, 93.00]	74.00 [53.00, 96.00]	72.00 [48.00,92.00]	74 [60.00, 93.00]	
HbA1c (%)		8.80 [7.10, 10.90]	8.40 [7.20, 10.70]	8.90 [7.00, 10.95]	8.70 [6.50, 10.60]	8.90 [7.60, 10.70]	0.713
Fasting C‐peptide (ng/mL)		1.73 [1.00, 2.94]	1.83 [1.30, 3.04]^d^	2.17 [1.26, 3.01]^d^	1.88 [0.82, 3.07]	1.22 [0.72, 1.70]^a^ ^,^ ^b^	0.004
HOMA‐IR		1.51 [1.50, 1.51]	1.51 [1.50, 1.51]^d^	1.51 [1.50, 1.51]	1.51 [1.50, 1.51]	1.50 [1.50, 1.51]^a^	0.003
eGFR (ml/min/1.73 m^2^)		95.1 [77.6, 102.7]	78.6 [66.4, 96.1]^b^ ^,^ ^d^	95.6 [83.6, 102.4]^a^	91.3 [67.5, 101.3]^d^	100.8 [93.3105.3]^a^ ^,^ ^c^	< 0.001

*Nutritional and inflammatory markers*							
Hemoglobin (g/L)		136.68 (17.01)	129.62 (15.84)^b^ ^,^ ^d^	141.38 (15.65)^a^ ^,^ ^c^	127.25 (19.08)^b^ ^,^ ^d^	138.90 (15.65)^a^ ^,^ ^c^	< 0.001
hsCRP (mg/L)		1.05 [0.49, 3.08]	2.22 [0.64, 4.84]^d^	1.21 [0.61, 3.07]^d^	1.03 [0.38, 3.01]	0.65 [0.44, 1.30]^a^ ^,^ ^b^	0.003

*Note:* HbA1c, glycosylated hemoglobin; HOMA‐IR, homeostasis‐model assessment for insulin resistance; hsCRP, hypersensitive C‐reactive protein.

Abbreviation: eGFR, estimated glomerular filtration ratio.

^a^Significantly different from the dynapenic obesity group.

^b^Significantly different from the nondynapenic obesity group.

^c^Significantly different from the dynapenic nonobesity group.

^d^Significantly different from the nondynapenic nonobesity group.

In terms of diabetes‐related features, the DO group presented increased rates of diabetic peripheral neuropathy (DPN) and hypertension, as well as higher DCSI scores and utilization of sodium‒glucose transport protein 2 (SGLT‐2) inhibitors and glucagon‐like peptide‐1 (GLP‐1) receptor agonist therapies, than did the NN group. The use of dipeptidyl peptidase‐4 (DPP‐4) inhibitors was less common in the DO group. However, there were no significant differences in the duration of diabetes; incidence of CHD, stroke, diabetic retinopathy (DR), diabetic nephropathy, or hypoglycemic events; or the use of metformin, insulin secretagogues, thiazolidinediones, insulin, statins, or renin‒angiotensin system (RAS) blockers between the two groups. Similarly, patients in the NO group more frequently chose medications that are more beneficial for both body weight management and positive prognosis, such as SGLT‐2 inhibitors and GLP‐1 receptor agonists, and they had a higher incidence of hypertension and a lower rate of DPP‐4 inhibitor usage than patients in the NN group. Moreover, the DN group had a longer duration of diabetes, a higher incidence of diabetic nephropathy, higher DCSI scores, and a higher rate of metformin usage (Table 2 and Supporting Table B).

**TABLE 2:** The diabetes‐related features, muscle function and anthropometry, and scale assessment of participants.

	**Category**	**Overall**	**Dynapenic obesity**	**Nondynapenic obesity**	**Dynapenic nonobesity**	**Nondynapenic nonobesity**	***p***
***n* (%)**		**270 (100.0)**	**56 (20.7)**	**121 (44.8)**	**32 (11.6)**	**61 (22.6)**	

Diabetes duration (years)		10.0 [6.0, 18.0]	14.0 [8.8, 20.0]	10.0 [4.0, 19.0]	15.0 [9.8, 19.5]^d^	10.0 [5.0, 13.0]^c^	0.018
Hypertension (%)		166 (61.9)	44 (78.6)^d^	75 (63.0)^d^	22 (68.8)	25 (41.0)^a^ ^,^ ^b^	< 0.001

*Complications*							
DR (%)		86 (32.1)	26 (46.4)^b^	29 (24.4)^a^ ^,^ ^c^	16 (50.0)^b^	15 (24.6)	0.002
DPN (%)		162 (60.7)	41 (73.2)^d^	72 (61.0)	21 (65.6)	28 (45.9)^a^	0.022
DCSI scores		4.00 [3.00, 5.00]	4.00 [3.00, 5.00]^d^	4.00 [3.00, 5.00]	4.50 [3.00, 6.00]^d^	3.00 [3.00, 4.00]^a^ ^,^ ^c^	0.005

*Medication*							
DPP‐4 inhibitors (%)		61 (22.8)	9 (16.1)^d^	21 (17.6)^d^	7 (21.9)	24 (39.3)^a^ ^,^ ^b^	0.005
SGLT‐2 inhibitors (%)		149 (55.6)	38 (67.9)^d^	73 (61.3)^d^	15 (46.9)	23 (37.7)^a^ ^,^ ^b^	0.003
GLP‐1 RA (%)		66 (24.8)	19 (34.5)^d^	40 (33.9)^c^ ^,^ ^d^	3 (9.4)^b^	4 (6.6)^a^ ^,^ ^b^	< 0.001

*Muscle function*							
Gait speed (m/s)		1.07 [0.87, 1.23]	0.91 [0.75, 1.06]^b^ ^,^ ^d^	1.10 [0.93, 1.28]^a^ ^,^ ^c^	0.94 [0.72, 1.15]^b^ ^,^ ^d^	1.15 [1.03, 1.28]^a^ ^,^ ^c^	< 0.001
Grip strength (kg)		25.8 [19.6, 33.0]	17.2 [14.8, 21.6]^b^ ^,^ ^d^	30.7 [22.0, 35.8]^a^ ^,^ ^c^	18.4 [14.6, 25.7]^b^ ^,^ ^d^	32.7 [28.3, 35.9]^a^ ^,^ ^c^	< 0.001

*Anthropometry*							
ASMI (kg/m^2^)		6.48 (1.03)	6.39 (0.81)	6.72 (1.06)^c^	5.77 (1.00)^b^ ^,^ ^d^	6.48 (0.97)^c^	0.001
ASM/W		0.27 [0.24, 0.29]	0.25 [0.23,0.27]^b^ ^,^ ^c^ ^,^ ^d^	0.27 [0.24, 0.29]^a^ ^,^ ^d^	0.28 [0.25, 0.30]^a^	0.30 [0.28, 0.31]^a^ ^,^ ^b^	< 0.001
BMI (kg/m^2^)		24.0 [22.4, 26.1]	25.7 [23.8, 27.0]^c^ ^,^ ^d^	25.1 [23.4, 26.6]^c^ ^,^ ^d^	21.5 [19.0, 22.6]^a^ ^,^ ^b^	22.4 [20.8, 23.7]^a^ ^,^ ^b^	< 0.001
WC (cm)		89.5 [83.5, 95.0]	93.8 [89.9, 99.5]^c^ ^.^ ^d^	92.5 [90.0, 97.0]^c^ ^,^ ^d^	78.5 [69.5, 84.5]^a^ ^,^ ^b^	84.0 [78.0, 86.5]^a^ ^,^ ^b^	< 0.001
BF% (%)		30.13 (6.13)	33.01 (5.72)^c^ ^,^ ^d^	31.63 (5.57)^c^ ^,^ ^d^	27.18 (5.83)^a^ ^,^ ^b^	26.07 (4.99)^a^ ^,^ ^b^	< 0.001
Limb fat mass (kg)		6.99 [5.99, 8.66]	8.62 [6.79, 9.81]^c^ ^,^ ^d^	7.57 [6.73, 9.06]^c^ ^,^ ^d^	5.72 [4.90, 6.33]^a^ ^,^ ^b^	6.07 [5.41, 6.78]^a^ ^,^ ^b^	< 0.001
eVATA (cm^2^)		123.52 (43.53)	144.02 (41.65)^c^ ^,^ ^d^	140.56 (36.49)^c^ ^,^ ^d^	76.80 (32.71)^a^ ^,^ ^b^	95.41 (27.24)^a^ ^,^ ^b^	< 0.001

*Scales*							
MoCA scores		25.0 [21.0, 27.0]	22.0 [17.0, 26.0]^d^ ^,^ ^b^	25.0 [22.0, 27.0]^a^	23.5 [19.8, 26.0]	26.0 [23.0, 27.0]^a^	< 0.001
MCI (%)		158 (58.5)	41 (73.2)^d^	66 (54.5)	22 (68.8)	29 (47.5)^a^	0.017
Depression (%)	Mild	25 (9.4)	7 (13.0)	11 (9.2)	2 (6.2)^d^	5 (8.2)^c^	0.023
	Moderate‐severe	22 (8.3)	4 (7.4)	10 (8.4)	6 (18.8)	2 (3.3)	
	Severe	4 (1.5)	1 (1.9)	0 (0.0)	3 (9.4)	0 (0.0)	
Sleep quality (%)	Fair	70 (26.1)	10 (18.5)^d^	29 (24.0)	7 (21.9)	24 (39.3)^a^	0.012
	Good	116 (43.3)	20 (37.0)	57 (47.1)	14 (43.8)	25 (41.0)	
	Poor	82 (30.6)	24 (44.4)	35 (28.9)	11 (34.4)	12 (19.7)	

*Note:* DPP‐4, dipeptidyl peptidase‐4; SGLT‐2, sodium–glucose transport protein 2; ASMI, appendicular skeletal muscle mass index; ASM/W, ratio of appendicular skeletal muscle mass to body weight; BF%, total body fat percentage; MoCA, Montreal Cognitive Assessment.

Abbreviations: BMI, body mass index; DCSI, Diabetes Complications Severity Index; DPN, diabetic peripheral neuropathy; DR, diabetic retinopathy; eVATA, estimated visceral adipose tissue area; GLP‐1 RA, glucagon‐like peptide‐1 receptor agonist; MCI, mild cognitive impairment; WC, waist circumference.

^a^Significantly different from the dynapenic obesity group.

^b^Significantly different from the nondynapenic obesity group.

^c^Significantly different from the dynapenic nonobesity group.

^d^Significantly different from the nondynapenic nonobesity group.

With respect to muscle function and anthropometry, both the DO and DN groups presented lower gait speeds than the NO and NN groups. BMI, BF%, limb fat mass, and eVATA were greater in the DO and NO groups than in the DN and NN groups. The ASMI of the DN group was lower than that of the NO and NN groups, whereas the ASMI of the DO group did not significantly differ from those of the other three groups. However, the ASM/W of the DO group was lower than that of the other three groups (Table 2).

### 3.2. Cognitive Function, Depression, and Sleep Conditions of the Participants

Compared with the NO and NN groups, the DO group had lower MoCA scores. Furthermore, the incidence rates of MCI in the DO, NO, DN, and NN groups were 73.2%, 54.5%, 68.8%, and 47.5%, respectively, with significant differences existing between the DO and NN groups. In terms of depression and sleep, the DO group performed worse than the NN group did, although this difference was not significant in terms of depression. Similarly, the level of depression in the DN group was also greater than that in the NN group (Table 2).

### 3.3. Association Between DAO and MoCA Scores

The relationship between the DAO and MoCA scores is presented in Table 3. Compared with the NN group, the DO group was associated with lower MoCA scores in Model 1 (B [95% CI], −4.00 [−5.81, −2.19], *p* < 0.001), Model 2 (B [95% CI], −2.07 [−4.11, −0.02], *p* = 0.048), and Model 3 (B [95% CI], −2.07 [−4.14, −0.01], *p* = 0.049). However, the difference was not observed between the NO or DN group and the NN group.

**TABLE 3:** Association between dynapenic abdominal obesity, grip strength, or waist circumference and MoCA scores in the linear regression analysis.

	**Model 1**			**Model 2**			**Model 3**		
	**R** ^2^	***B* (95% CI)**	***P***	**R** ^2^	***B* (95% CI)**	***P***	**R** ^2^	***B* (95% CI)**	***P***

Nondynapenic nonobesity	Reference			Reference			Reference		
Dynapenic nonobesity	0.082	−2.69 (−4.46, −0.93)	**0.003**	0.271	−1.73 (−3.50, 0.05)	0.057	0.289	−1.11 (−2.96, 0.74)	0.235
Nondynapenic obesity	0.005	−0.90 (−2.17, 0.37)	0.162	0.225	−1.09 (−2.28, 0.10)	0.073	0.228	−1.01 (−2.23, 0.21)	0.103
Dynapenic obesity	0.136	−4.00 (−5.81, −2.19)	**< 0.001**	0.182	−2.06 (−4.11, −0.02)	**0.048**	0.218	−2.07 (−4.14, −0.01)	**0.049**

Grip strength (kg)	0.118	0.19 (0.13, 0.25)	**< 0.001**	0.220	0.15 (0.06, 0.25)	**0.002**	0.239	0.14 (0.05, 0.24)	**0.004**
Waist circumference (cm)	−0.003	−0.01 (−0.06, 0.05)	0.755	0.201	−0.05 (−0.11, 0.00)	**0.043**	0.224	−0.05 (−0.10, 0.00)	0.059

*Note:* The bold values indicate statistically significant *p* values (i.e., *p* < 0.05). MoCA, Montreal Cognitive Assessment; B, B coefficients. Model 1: unadjusted. Model 2: adjusted for age, sex, smoking status, alcohol intake, education level, income, living alone, and depression. Model 3: adjusted for Model 2 + glycosylated hemoglobin and Diabetes Complications Severity Index scores.

Abbreviation: CI, confidence interval.

GS was significantly positively correlated with MoCA scores in all three models (Model 1, B [95% CI], 0.19 [0.13, 0.25], *p* < 0.001; Model 2, B [95% CI], 0.15 [0.06, 0.25], *p* = 0.002; Model 3, B [95% CI], 0.14 [0.05, 0.24], *p* = 0.004). However, WC was negatively correlated with the MoCA score only in Model 2 (B [95% CI], −0.05 [−0.11, 0.00], *p* = 0.043) (Table 3).

### 3.4. Influencing Factors for DAO

**FIGURE 2:**
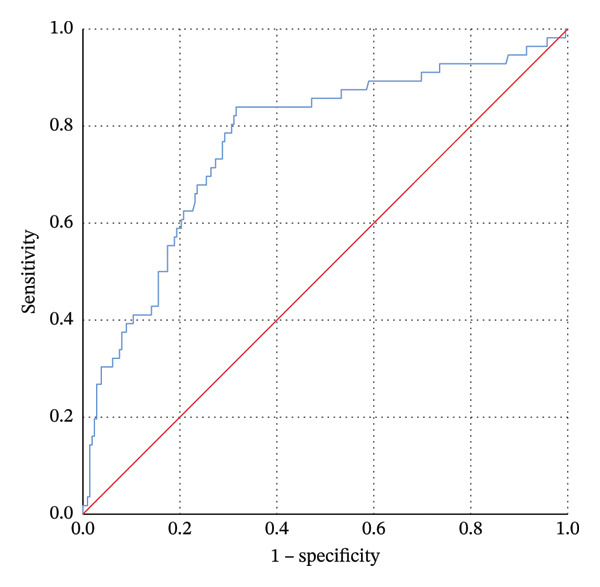
The receiver operating characteristic (ROC) curve for predicting dynapenic abdominal obesity using age, gait speed, and diabetic retinopathy. Area under the curve (AUC): 0.768 (95% CI: 0.694, 0.842). Maximum Youden’s index, 0.523; corresponding cutoff value of 0.2067; sensitivity of 83.9%; and specificity of 68.4%.

As previously mentioned, we selected the independent variables for DAO with *p* < 0.1, including age, sex, income, educational level, gait speed, hemoglobin, diabetes duration, DPN, and DR, and ultimately included them in the multivariate logistic regression. The following factors are associated with DAO: age (odds ratio [OR] = 1.077, *p* = 0.001), gait speed (OR = 0.109, *p* < 0.001) and DR (OR = 2.475, *p* = 0.008) (Table 4). A predictive model was subsequently constructed based on these factors, and the ROC curve was plotted. The area under the curve (AUC) was 0.768 (95% CI: 0.694–0.842), with a maximum Youden’s index of 0.523, corresponding to a cutoff value of 0.2067, a sensitivity of 83.9%, and a specificity of 68.4% (Figure 2).

**TABLE 4:** Influencing factors for dynapenic abdominal obesity via multivariate logistic regression.

Variables	*B*	OR (95% CI)	*P*
Age (years)	0.074	1.077 (1.029, 1.126)	0.001
Gait speed (m/s)	−2.220	0.109 (0.032, 0.365)	< 0.001
Diabetic retinopathy	0.906	2.475 (1.273, 4.815)	0.008

*Note:* B, B coefficients. Include age, sex, income, education level, gait speed, hemoglobin, diabetes duration, diabetic peripheral neuropathy, and diabetic retinopathy.

Abbreviations: CI, confidence interval; OR, odds ratio.

## 4. Discussion

Diabetes represents a grave societal challenge intricately linked with a diverse spectrum of disorders, notably DAO and the deterioration of cognitive functions. This study revealed that DAO is relatively common among middle‐aged and older individuals with T2D and usually presents unique traits related to age and diabetes. More importantly, the presence of DAO appears to be robustly linked to poorer cognitive function, and such a correlation remains significant even after adjusting for key confounding variables, suggesting that DAO, for the first time, has been recognized within diabetic sufferers as a simple, user‐friendly, and modifiable marker that may play a potential role in potentially preventing and delaying adverse cognitive outcomes.

To date, most studies on obesity have concentrated on overall or regional fat. Few have focused on the interplay between excessive adipose accumulation and loss of skeletal muscle mass and function, although such interactions may constitute a vicious cycle. Our data appear to support this integrated idea for DAO, since in the present study, the DAO patients displayed a reduced relative muscle mass alongside an increase in visceral and limb adiposity. Moreover, they also exhibit more severe insulin resistance and higher‐grade inflammation, coupled with poorer nutritional status, making them more prone to diabetes complications and comorbidities, with a greater diabetic burden. The mechanism for the cycle between components of DAO is still unclear. Earlier studies have shown that obesity can trigger chronic inflammation, which may result in muscle atrophy [25]. Concurrently, excessive free fatty acids during obesity can lead to insulin resistance and changes in the secretion of proinflammatory myokines, ultimately contributing to a reduction in muscle mass [26]. On the other hand, alterations in the release of proinflammatory myokines are closely related to the poor browning response of white adipose tissue [27], thereby causing an increase in fat. Additionally, a reduction in muscle mass may also lead to a decrease in physical activity and energy expenditure, further exacerbating obesity. However, whether the formation of DAO in the diabetic population is mediated by such complex interactions still requires further research.

As is the case for other geriatric syndromes, DAO often occurs when multiple domains are impaired. Our results also indicated that age, slow gait, and DR are independent influencing factors for DAO. This finding is consistent with previous research. Baarts et al. reported that as age advances, there is an accumulation of visceral fat, coupled with a decline in both muscle mass and functionality [28, 29]. Additionally, an 8‐year longitudinal study demonstrated that DAO was strongly correlated with reduced gait speed [11]. Moreover, a meta‐analysis revealed a significant correlation between diabetic complications and an increased possibility of developing sarcopenia [30]. Another prospective study by Chen et al. revealed that obesity is related to an increased risk of diabetic microvascular complications [31]. Abnormal differentiation of stem cells may be a monistic explanation for the above correlation. Aging and changes in unhealthy lifestyles or metabolic microenvironments tend to promote mesenchymal stem cell differentiation into adipocytes as opposed to myocytes and endothelial cells, which may lead to fat accumulation, muscle atrophy, and endothelial cell dysfunction [32, 33].

Metabolic disturbance, functional disability, and cardiovascular disease have long been recognized as negative outcomes of DAO; however, our analysis revealed that among T2D patients, DAO is independently associated with cognitive impairment, which is similar to the findings of Oba et al. in a high‐risk frail population [14]. DAO and cognitive impairment are components of geriatric syndrome, with aging serving as the common biological backdrop for both [34]. On the other hand, metabolic disorders and chronic inflammation, as key pathophysiological bases of DAO, may also have a negative impact on cognitive function. Insulin resistance is associated with the deposition of amyloid‐β (Aβ) and tau proteins in the brain, which are the histopathological hallmarks of Alzheimer’s disease. Additionally, neuroinflammation in brain regions is linked with cognitive impairment [35]. Moreover, we discovered that a decrease in GS is independently correlated with cognitive impairment, which is consistent with the conclusions of several prospective studies [36]. However, no association was found between an increase in WC and cognitive function. Although studies have indicated that central obesity is significantly related to a reduction in cognitive function, this correlation is more pronounced in individuals with normal weight, and central obesity is identified by an increased waist‐to‐hip ratio rather than WC [37, 38]. Nevertheless, other prospective studies have shown no statistically significant association between WC and cognitive disorders [39]. More studies are needed to further clarify the relationship between WC and cognitive function.

This study has several limitations. First, it is a cross‐sectional study, and causality cannot be determined. In addition, this was a single‐center study with a small sample size, and further research is needed across a more diverse racial spectrum, within a nondiabetic population, and with an expanded sample size to confirm our conclusions.

## 5. Conclusions

In summary, our results indicate that DAO is relatively common among middle‐aged and older patients with T2D and clearly displays unique features linked to age and diabetes. Compared with abdominal obesity or dynapenia alone, DAO is significantly associated with an increased risk of cognitive impairment, even after adjusting for diverse confounders. More research is needed to explore whether cognitive decline can be prevented or delayed by improving body composition in subjects with or without diabetes.

## Author Contributions

Conceptualization: Bo Zhou; methodology: Bo Zhou; formal analysis and investigation: Yinghao Li, Yan Pi, Xin Ye, Kun Liao, and Fengning Chuan; writing–original draft preparation: Yinghao Li and Yan Pi; writing–review and editing: Bo Zhou; resources: Mei Mei and Rong Li; supervision: Yinghao Li.

## Funding

This research did not receive any specific grant from funding agencies in the public, commercial, or not‐for‐profit sectors.

## Ethics Statement

This study protocol conformed to the Declaration of Helsinki and was approved by the Ethics Committee of the First Affiliated Hospital of Chongqing Medical University (No. K2024‐112‐01).

## Conflicts of Interest

The authors declare no conflicts of interest.

## Supporting Information

Supporting Table A. The appendant demographics and biochemical indicators of participants.

Supporting Table B. The appendant diabetes‐related features of participants.

STROBE Statement—checklist of items that should be included in reports of observational studies.

## Supporting information


**Supporting Information** Additional supporting information can be found online in the Supporting Information section.

## Data Availability

The datasets generated during and/or analyzed during the current study are available from the corresponding author on reasonable request.

## References

[1] Borghesan M. , Hoogaars W. M. H. , Varela-Eirin M. , Talma N. , and Demaria M. , A Senescence-Centric View of Aging: Implications for Longevity and Disease, Trends in Cell Biology. (2020) 30, no. 10, 777–791, 10.1016/j.tcb.2020.07.002. 32800659

[2] Sun H. , Saeedi P. , Karuranga S. et al., IDF Diabetes Atlas: Global, Regional and Country-Level Diabetes Prevalence Estimates for 2021 and Projections for 2045, Diabetes Research and Clinical Practice. (2023) 204, 10.1016/j.diabres.2023.110945. PMC11057359 34879977

[3] Deng M. Q. , Pan Q. , Xiao X. H. , and Guo L. X. , [Interpretations of Guideline for the Management of Diabetes Mellitus in the Elderly in China (2021 Edition)], Zhonghua Nei Ke Za Zhi. (2021) 60, no. 11, 954–959, Chinese 10.3760/cma.j.cn112138-20210305-00183. 34689515

[4] Xue M. , Xu W. , Ou Y. N. et al., Diabetes Mellitus and Risks of Cognitive Impairment and Dementia: A Systematic Review and Meta-Analysis of 144 Prospective Studies, Ageing Research Reviews. (2019) 55, 10.1016/j.arr.2019.100944, 2-s2.0-85071229063. 31430566

[5] Biessels G. J. and Whitmer R. A. , Cognitive Dysfunction in Diabetes: How to Implement Emerging Guidelines, Diabetologia. (2020) 63, no. 1, 3–9, 10.1007/s00125-019-04977-9, 2-s2.0-85071048858. 31420699 PMC6890615

[6] Livingston G. , Huntley J. , Sommerlad A. et al., Dementia Prevention, Intervention, and Care: 2020 Report of the Lancet Commission, Lancet. (2023) 402, no. 10408, 10.1016/S0140-6736(23)02043-3. PMC7392084 32738937

[7] Hou Q. , Guan Y. , Yu W. et al., Associations Between Obesity and Cognitive Impairment in the Chinese Elderly: An Observational Study, Clinical Interventions in Aging. (2019) 14, 367–373, 10.2147/CIA.S192050, 2-s2.0-85062704446. 30863030 PMC6388776

[8] Qizilbash N. , Gregson J. , Johnson M. E. et al., BMI and Risk of Dementia in Two Million People Over Two Decades: A Retrospective Cohort Study, Lancet Diabetes & Endocrinology. (2015) 3, no. 6, 431–436, 10.1016/S2213-8587(15)00033-9, 2-s2.0-84929954141. 25866264

[9] Burns J. M. , Johnson D. K. , Watts A. , Swerdlow R. H. , and Brooks W. M. , Reduced Lean Mass in Early Alzheimer Disease and Its Association With Brain Atrophy, Archives of Neurology. (2010) 67, no. 4, 428–433, 10.1001/archneurol.2010.38, 2-s2.0-77950911528. 20385908 PMC2855150

[10] Sui S. X. , Williams L. J. , Holloway-Kew K. L. , Hyde N. K. , and Pasco J. A. , Skeletal Muscle Health and Cognitive Function: A Narrative Review, International Journal of Molecular Sciences. (2020) 22, no. 1, 10.3390/ijms22010255. PMC7795998 33383820

[11] de Oliveira Máximo R. , de Oliveira D. C. , Ramírez P. C. et al., Dynapenia, Abdominal Obesity or Both: Which Accelerates the Gait Speed Decline Most?, Age and Ageing. (2021) 50, no. 5, 1616–1625, 10.1093/ageing/afab093. 34087934 PMC8437070

[12] Sabag A. , Way K. L. , Keating S. E. et al., Exercise and Ectopic Fat in Type 2 Diabetes: A Systematic Review and Meta-Analysis, Diabetes & Metabolism. (2017) 43, no. 3, 195–210, 10.1016/j.diabet.2016.12.006, 2-s2.0-85011296324. 28162956

[13] Ramírez P. C. , de Oliveira Máximo R. , Capra de Oliveira D. et al., Dynapenic Abdominal Obesity as a Risk Factor for Metabolic Syndrome in Individual 50 Years of Age or Older: English Longitudinal Study of Ageing, The Journal of Nutrition, Health & Aging. (2023) 27, no. 12, 1188–1195, 10.1007/s12603-023-2039-1. PMC12880461 38151869

[14] Oba K. , Tamura Y. , Ishikawa J. et al., Dynapenic Abdominal Obesity is Associated With Mild Cognitive Impairment in Patients With Cardiometabolic Disease: A Cross-Sectional Study, BMC Geriatrics. (2022) 22, no. 1, 10.1186/s12877-022-02948-1. PMC8962154 35346081

[15] World Health Organization , Definition, Diagnosis and Classification of Diabetes Mellitus and Its Complications: Report of a WHO Consultation, Part 1. Diagnosis and Classification of Diabetes Mellitus. (1999) World Health Organization, Geneva.

[16] Chen L. K. , Woo J. , Assantachai P. et al., Asian Working Group for Sarcopenia: 2019 Consensus Update on Sarcopenia Diagnosis and Treatment, Journal of the American Medical Directors Association. (2020) 21, no. 3, 300–307.e2, 10.1016/j.jamda.2019.12.012. 32033882

[17] Chinese Nutrition Society Obesity Prevention and Control Section; Chinese Nutrition Society Clinical Nutrition Section , Chinese Preventive Medicine Association Behavioral Health Section; Chinese Preventive Medicine Association Sports and Health Section. [Expert Consensus on Obesity Prevention and Treatment in China], Zhonghua Liuxingbingxue Zazhi. (2022) 43, no. 5, 609–626, Chinese 10.3760/cma.j.cn112338-20220402-00253. 35589563

[18] Chinese Society of Dementia and Cognitive Impairment , Chen X. C. , Pan X. D. et al., [Chinese Expert Consensus on the Diagnosis and Treatment of Mild Cognitive Impairment due to Alzheimer′S Disease 2021], Zhonghua Shen Jing Ke Za Zhi. (May 2022) 55, no. 5, 421–440, Chinese.

[19] Li X. , Zhou Z. G. , Qi H. Y. , Chen X. Y. , and Huang G. , [Replacement of Insulin by Fasting C-Peptide in Modified Homeostasis Model Assessment to Evaluate Insulin Resistance and Islet Beta Cell Function], Zhong Nan Da Xue Xue Bao Yi Xue Ban. (2004) 29, no. 4, 419–423, Chinese. 16134594

[20] Young B. A. , Lin E. , Von Korff M. et al., Diabetes Complications Severity Index and Risk of Mortality, Hospitalization, and Healthcare Utilization, American Journal of Managed Care. (2008) 14, no. 1, 15–23. 18197741 PMC3810070

[21] Workgroup on Hypoglycemia and American Diabetes Association , Defining and Reporting Hypoglycemia in Diabetes: A Report from the American Diabetes Association Workgroup on Hypoglycemia, Diabetes Care. (2005) 28, no. 5, 1245–1249, 10.2337/diacare.28.5.1245, 2-s2.0-18144388647. 15855602

[22] Craig C. L. , Marshall A. L. , Sjöström M. et al., International Physical Activity Questionnaire: 12-Country Reliability and Validity, Medicine & Science in Sports & Exercise. (2003) 35, no. 8, 1381–1395, 10.1249/01.MSS.0000078924.61453.FB, 2-s2.0-0042855872. 12900694

[23] Kroenke K. , Spitzer R. L. , and Williams J. B. , The PHQ-9: Validity of a Brief Depression Severity Measure, Journal of General Internal Medicine. (2001) 16, no. 9, 606–613, 10.1046/j.1525-1497.2001.016009606.x, 2-s2.0-0034853189. 11556941 PMC1495268

[24] Buysse D. J. , Reynolds C. F. , Monk T. H. , Berman S. R. , and Kupfer D. J. , The Pittsburgh Sleep Quality Index: A New Instrument for Psychiatric Practice and Research, Psychiatry Research. (1989) 28, no. 2, 193–213, 10.1016/0165-1781(89)90047-4, 2-s2.0-0024389366. 2748771

[25] Schrager M. A. , Metter E. J. , Simonsick E. et al., Sarcopenic Obesity and Inflammation in the InCHIANTI Study, Journal of Applied Physiology. (2007) 102, no. 3, 919–925, 10.1152/japplphysiol.00627.2006, 2-s2.0-33847767040. 17095641 PMC2645665

[26] Kalinkovich A. and Livshits G. , Sarcopenic Obesity or Obese Sarcopenia: A Cross Talk Between Age-Associated Adipose Tissue and Skeletal Muscle Inflammation as a Main Mechanism of the Pathogenesis, Ageing Research Reviews. (2017) 35, 200–221, 10.1016/j.arr.2016.09.008, 2-s2.0-85005801887. 27702700

[27] Boström P. , Wu J. , Jedrychowski M. P. et al., A PGC1-α-Dependent Myokine That Drives Brown-Fat-Like Development of White Fat and Thermogenesis, Nature. (2012) 481, no. 7382, 463–468, 10.1038/nature10777, 2-s2.0-84862776702. 22237023 PMC3522098

[28] Baarts R. B. , Jensen M. R. , Hansen O. M. et al., Age- and Sex-Specific Changes in Visceral Fat Mass Throughout the Life-Span, Obesity. (2023) 31, no. 7, 1953–1961, 10.1002/oby.23779. 37312268

[29] Charlier R. , Knaeps S. , Mertens E. et al., Age-Related Decline in Muscle Mass and Muscle Function in Flemish Caucasians: A 10-Year Follow-Up, Age. (2016) 38, no. 2, 10.1007/s11357-016-9900-7, 2-s2.0-84960942535. PMC5005902 26961694

[30] Qiao Y. S. , Chai Y. H. , Gong H. J. et al., The Association Between Diabetes Mellitus and Risk of Sarcopenia: Accumulated Evidences From Observational Studies, Frontiers in Endocrinology. (2021) 12, 10.3389/fendo.2021.782391. PMC8734040 35002965

[31] Chen J. , Li Y. T. , Niu Z. et al., Investigating the Causal Association of Generalized and Abdominal Obesity With Microvascular Complications in Patients With Type 2 Diabetes: A Community-Based Prospective Study, Diabetes, Obesity and Metabolism. (2024) 26, no. 7, 2796–2810, 10.1111/dom.15598. 38695216

[32] Yang X. , Wang Y. , Rovella V. et al., Aged Mesenchymal Stem Cells and Inflammation: From Pathology to Potential Therapeutic Strategies, Biology Direct. (2023) 18, no. 1, 10.1186/s13062-023-00394-6. PMC10353240 37464416

[33] Sahinyan K. , Lazure F. , Blackburn D. M. , and Soleimani V. D. , Decline of Regenerative Potential of Old Muscle Stem Cells: Contribution to Muscle Aging, FEBS Journal. (2023) 290, no. 5, 1267–1289, 10.1111/febs.16352. 35029021

[34] Neumiller J. J. and Munshi M. N. , Geriatric Syndromes in Older Adults With Diabetes, Endocrinology and Metabolism Clinics of North America. (2023) 52, no. 2, 341–353, 10.1016/j.ecl.2022.10.004. 36948783

[35] Arnoriaga-Rodríguez M. and Fernández-Real J. M. , Microbiota Impacts on Chronic Inflammation and Metabolic Syndrome-Related Cognitive Dysfunction, Reviews in Endocrine & Metabolic Disorders. (2019) 20, no. 4, 473–480, 10.1007/s11154-019-09537-5. 31884557

[36] McGrath R. , Robinson-Lane S. G. , Cook S. et al., Handgrip Strength Is Associated With Poorer Cognitive Functioning in Aging Americans, Journal of Alzheimer’s Disease. (2019) 70, no. 4, 1187–1196, 10.3233/JAD-190042, 2-s2.0-85071268841. PMC9483826 31322562

[37] Kerwin D. R. , Gaussoin S. A. , Chlebowski R. T. et al., Interaction Between Body Mass Index and Central Adiposity and Risk of Incident Cognitive Impairment and Dementia: Results From the Women’s Health Initiative Memory Study, Journal of the American Geriatrics Society. (2011) 59, no. 1, 107–112, 10.1111/j.1532-5415.2010.03219.x, 2-s2.0-78651403233. 21226681

[38] Cho G. J. , Hwang S. Y. , Lee K. M. et al., Association Between Waist Circumference and Dementia in Older Persons: A Nationwide Population-Based Study, Obesity. (2019) 27, no. 11, 1883–1891, 10.1002/oby.22609. 31689005

[39] Ren Z. , Li Y. , Li X. et al., Associations of Body Mass Index, Waist Circumference and Waist-to-Height Ratio With Cognitive Impairment Among Chinese Older Adults: Based on the CLHLS, Journal of Affective Disorders. (2021) 295, 463–470, 10.1016/j.jad.2021.08.093. 34507227

