# Supplementary material for: Dynapenic Abdominal Obesity and Cognitive Impairment in Type 2 Diabetic Patients: A Single‐Center Cross‐Sectional Study
Source: Int J Endocrinol. 2026 Mar 12;2026:6060666. doi: 10.1155/ije/6060666 (PMC13098355; doi:10.1155/ije/6060666)
Supplement: Supplementary file 1 — Supporting Information Additional supporting information can be found online in the Supporting Information section. [file IJE-2026-6060666-s001.zip › STROBE-checklist-v4-combined-PlosMedicine.docx]

STROBE Statement—checklist of items that should be included in reports of observational studies

|  | Item No. | Recommendation | Page  No. | Relevant text from manuscript |
| --- | --- | --- | --- | --- |
| **Title and abstract** | 1 | (*a*) Indicate the study’s design with a commonly used term in the title or the abstract | 1 | A Single-Center Cross-Sectional Study |
|  |  | (*b*) Provide in the abstract an informative and balanced summary of what was done and what was found | 2 | Among middle-aged and older individuals with T2D, DAO is independently associated with cognitive impairment and presents phenotypic features linked to age and diabetes. |
| Introduction | | | |  |
| Background/rationale | 2 | Explain the scientific background and rationale for the investigation being reported | 5 | A cross-sectional study published in 2022 demonstrated that DAO is significantly correlated with mild cognitive impairment (MCI) in patients with cardiometabolic disease; however, very few studies have explored the relationship between DAO and cognition among the population with diabetes, to date. |
| Objectives | 3 | State specific objectives, including any prespecified hypotheses | 5 | Therefore, to determine the role of DAO as a surrogate marker for cognitive outcome, this study first investigated the phenotypic characteristics of patients with DAO among middle-aged and older patients with T2D. The relationship between DAO and cognitive impairment was subsequently observed after adjusting for potential confounders. Finally, we analyzed whether one of the two components of DAO, namely, grip strength (GS) or waist circumference (WC), is independently associated with cognitive impairment. |
| Methods | | | |  |
| Study design | 4 | Present key elements of study design early in the paper | 11 | The association between DAO and MoCA scores were evaluated via multiple linear regression. Three models were designed: Model 1 was unadjusted; Model 2 was adjusted for age, sex, smoking status, alcohol intake, education level, income, living alone and being depressed; and Model 3 was further adjusted for DCSI and HbA1c. Additionally, multiple linear regression was conducted to explore the relationship between GS or WC and MoCA scores. Finally, to explore the influencing factors of DAO, we treated DAO as a binary dependent variable and considered demographics, lifestyle habits, nutritional and inflammatory markers, and diabetes-related characteristics as independent variables, performing univariate logistic regression for each. Ultimately, the independent variables with P < 0.1 in the univariate logistic regression were included in the multivariate logistic regression, and the receiver operating characteristic (ROC) curves were plotted. |
| Setting | 5 | Describe the setting, locations, and relevant dates, including periods of recruitment, exposure, follow-up, and data collection | 5 | Participants were consecutively enrolled from the Department of Endocrinology, the First Affiliated Hospital of Chongqing Medical University between September 2020 and January 2024. |
| Participants | 6 | (*a*) *Cohort study*—Give the eligibility criteria, and the sources and methods of selection of participants. Describe methods of follow-up  *Case-control study*—Give the eligibility criteria, and the sources and methods of case ascertainment and control selection. Give the rationale for the choice of cases and controls  *Cross-sectional study*—Give the eligibility criteria, and the sources and methods of selection of participants | 5 | Participants were consecutively enrolled from the Department of Endocrinology, the First Affiliated Hospital of Chongqing Medical University between September 2020 and January 2024. The inclusion criteria were as follows: 1) previously diagnosed with T2D based on the 1999 World Health Organization diagnostic criteria; 2) aged ≥ 50 years; and 3) capable of self-care and communication in daily life. The exclusion criteria were as follows: 1) acute complications of diabetes, serious infections, surgery or other stress states; 2) diseases that significantly affect cognitive function, such as neurological disorders (Alzheimer's disease, vascular dementia, brain tumors, etc.), vitamin B12 or folate deficiency, hypothyroidism, hypercalcemia, etc.; 3) the use of medications that affect cognitive function, such as antipsychotics, antidepressants, benzodiazepines, or anticholinergic drugs, etc.; 4) severe organ dysfunction, cancer, or autoimmune diseases; 5) conditions affecting anthropometry or muscle function measurements, such as edema, limb amputation or injury, etc.; and 6) inability or unwillingness to cooperate with the study. Each participant completed questionnaires and scales, blood tests, and physical examinations. |
|  |  | (*b*) *Cohort study*—For matched studies, give matching criteria and number of exposed and unexposed  *Case-control study*—For matched studies, give matching criteria and the number of controls per case | Not applicable |  |
| Variables | 7 | Clearly define all outcomes, exposures, predictors, potential confounders, and effect modifiers. Give diagnostic criteria, if applicable | 11 | The association between DAO and MoCA scores were evaluated via multiple linear regression. Three models were designed: Model 1 was unadjusted; Model 2 was adjusted for age, sex, smoking status, alcohol intake, education level, income, living alone and being depressed; and Model 3 was further adjusted for DCSI and HbA1c. Additionally, multiple linear regression was conducted to explore the relationship between GS or WC and MoCA scores. Finally, to explore the influencing factors of DAO, we treated DAO as a binary dependent variable and considered demographics, lifestyle habits, nutritional and inflammatory markers, and diabetes-related characteristics as independent variables, performing univariate logistic regression for each. |
| Data sources/ measurement | 8* | For each variable of interest, give sources of data and details of methods of assessment (measurement). Describe comparability of assessment methods if there is more than one group | *9* | *The Montreal Cognitive Assessment (MoCA), with a total score of 30 points, was used to evaluate cognitive function. When the subject's years of education were ≤ 12 years, an additional point was added to the scores. Based on Chinese expert consensus on the diagnosis of MCI 2021, MCI was defined as a MoCA score < 26 points.* |
| Bias | 9 | Describe any efforts to address potential sources of bias | 11 | The association between DAO and MoCA scores were evaluated via multiple linear regression. Three models were designed: Model 1 was unadjusted; Model 2 was adjusted for age, sex, smoking status, alcohol intake, education level, income, living alone and being depressed; and Model 3 was further adjusted for DCSI and HbA1c. Additionally, multiple linear regression was conducted to explore the relationship between GS or WC and MoCA scores. Finally, to explore the influencing factors of DAO, we treated DAO as a binary dependent variable and considered demographics, lifestyle habits, nutritional and inflammatory markers, and diabetes-related characteristics as independent variables, performing univariate logistic regression for each. Ultimately, the independent variables with P < 0.1 in the univariate logistic regression were included in the multivariate logistic regression, and the receiver operating characteristic (ROC) curves were plotted. |
| Study size | 10 | Explain how the study size was arrived at | Not mentioned |  |

Continued on next page

| Quantitative variables | 11 | Explain how quantitative variables were handled in the analyses. If applicable, describe which groupings were chosen and why | 11 | The association between DAO and MoCA scores were evaluated via multiple linear regression. Three models were designed: Model 1 was unadjusted; Model 2 was adjusted for age, sex, smoking status, alcohol intake, education level, income, living alone and being depressed; and Model 3 was further adjusted for DCSI and HbA1c. Additionally, multiple linear regression was conducted to explore the relationship between GS or WC and MoCA scores. Finally, to explore the influencing factors of DAO, we treated DAO as a binary dependent variable and considered demographics, lifestyle habits, nutritional and inflammatory markers, and diabetes-related characteristics as independent variables, performing univariate logistic regression for each. Ultimately, the independent variables with P < 0.1 in the univariate logistic regression were included in the multivariate logistic regression, and the receiver operating characteristic (ROC) curves were plotted. |
| --- | --- | --- | --- | --- |
| Statistical methods | 12 | (*a*) Describe all statistical methods, including those used to control for confounding | 11 | The normality of the data was assessed via the Shapiro‒Wilk test. Continuous variables are expressed as the means (standard deviations, SDs) or medians (interquartile ranges, IQRs), whereas categorical variables are presented as numbers (%). One-way ANOVA or the Kruskal‒Wallis test was used to compare overall differences in continuous variables among the four groups, and post hoc comparisons were made via the LSD test or the Mann‒Whitney U test to assess differences between groups. The chi-square test or Fisher's exact test was used to compare differences in categorical variables among and between groups. P values were adjusted via the Bonferroni correction, and a two-sided P value < 0.05 was considered statistically significant. The association between DAO and MoCA scores were evaluated via multiple linear regression. Three models were designed: Model 1 was unadjusted; Model 2 was adjusted for age, sex, smoking status, alcohol intake, education level, income, living alone and being depressed; and Model 3 was further adjusted for DCSI and HbA1c. Additionally, multiple linear regression was conducted to explore the relationship between GS or WC and MoCA scores. Finally, to explore the influencing factors of DAO, we treated DAO as a binary dependent variable and considered demographics, lifestyle habits, nutritional and inflammatory markers, and diabetes-related characteristics as independent variables, performing univariate logistic regression for each. Ultimately, the independent variables with P < 0.1 in the univariate logistic regression were included in the multivariate logistic regression, and the receiver operating characteristic (ROC) curves were plotted. |
|  |  | (*b*) Describe any methods used to examine subgroups and interactions | 11 | The association between DAO and MoCA scores were evaluated via multiple linear regression. Three models were designed: Model 1 was unadjusted; Model 2 was adjusted for age, sex, smoking status, alcohol intake, education level, income, living alone and being depressed; and Model 3 was further adjusted for DCSI and HbA1c. |
|  |  | (*c*) Explain how missing data were addressed | Not mentioned |  |
|  |  | (*d*) *Cohort study*—If applicable, explain how loss to follow-up was addressed  *Case-control study*—If applicable, explain how matching of cases and controls was addressed  *Cross-sectional study*—If applicable, describe analytical methods taking account of sampling strategy | Not mentioned |  |
|  |  | (*e*) Describe any sensitivity analyses | Not mentioned |  |
| Results | | | | |
| Participants | 13* | (a) Report numbers of individuals at each stage of study—eg numbers potentially eligible, examined for eligibility, confirmed eligible, included in the study, completing follow-up, and analysed | 6 | The flowchart of the subjects is shown in Figure 1, ultimately resulting in 270 participants enrolled in this study. |
|  |  | (b) Give reasons for non-participation at each stage | 6 | The flowchart of the subjects is shown in Figure 1. |
|  |  | (c) Consider use of a flow diagram | 6 | The flowchart of the subjects is shown in Figure 1. |
| Descriptive data | 14* | (a) Give characteristics of study participants (eg demographic, clinical, social) and information on exposures and potential confounders | 12 | The clinical profiles of the participants, categorized by dynapenia status and abdominal obesity status, are displayed in Table 1 and Supplementary Table A. A total of 115 females and 155 males were included in the study, with an average age of 66.0 (59.0, 71.0) years. In total, 56 (20.7%), 121 (44.8%), 32 (11.6%), and 61 (22.6%) subjects were in the DO, NO, DN, and NN groups, respectively. |
|  |  | (b) Indicate number of participants with missing data for each variable of interest | Not applicable |  |
|  |  | (c) *Cohort study*—Summarise follow-up time (eg, average and total amount) | Not applicable |  |
| Outcome data | 15* | *Cohort study*—Report numbers of outcome events or summary measures over time | Not applicable |  |
|  |  | *Case-control study—*Report numbers in each exposure category, or summary measures of exposure | Not applicable |  |
|  |  | *Cross-sectional study—*Report numbers of outcome events or summary measures | *17* | *Compared with the NO and NN groups, the DO group had lower MoCA scores. Furthermore, the incidence rates of MCI in the DO, NO, DN, and NN groups were 73.2%, 54.5%, 68.8%, and 47.5%, respectively, with significant differences existing between the DO and NN groups.* |
| Main results | 16 | (*a*) Give unadjusted estimates and, if applicable, confounder-adjusted estimates and their precision (eg, 95% confidence interval). Make clear which confounders were adjusted for and why they were included | 18 | The relationship between the DAO and MoCA scores is presented in Table 3. Compared with the NN group, the DO group was associated with lower MoCA scores in Model 1 (B [95% CI], -4.00 [-5.81, -2.19], P < 0.001), Model 2 (B [95% CI], -2.07 [-4.11, -0.02], P = 0.048), and Model 3 (B [95% CI], -2.07 [-4.14, -0.01], P = 0.049). However, the difference was not observed between the NO or DN group and NN group. |
|  |  | (*b*) Report category boundaries when continuous variables were categorized | 7 | Based on Chinese expert consensus on the diagnosis of MCI 2021, MCI was defined as a MoCA score < 26 points. |
|  |  | (*c*) If relevant, consider translating estimates of relative risk into absolute risk for a meaningful time period | Not mentioned |  |

Continued on next page

| Other analyses | 17 | Report other analyses done—eg analyses of subgroups and interactions, and sensitivity analyses | 19 | As previously mentioned, we selected the independent variables for DAO with P < 0.1, including age, sex, income, educational level, gait speed, hemoglobin, diabetes duration, DPN and DR, and ultimately included them in the multivariate logistic regression. The following factors are associated with DAO: age (odds ratio [OR] = 1.077, P = 0.001), gait speed (OR = 0.109, P < 0.001) and DR (OR = 2.475, P = 0.008) (Table 4). A predictive model was subsequently constructed based on these factors, and the ROC curve was plotted. The area under the curve (AUC) was 0.768 (95% CI: 0.694–0.842), with a maximum Youden's index of 0.523, corresponding to a cutoff value of 0.2067, a sensitivity of 83.9%, and a specificity of 68.4% (Figure 2). |
| --- | --- | --- | --- | --- |
| Discussion | | | | |
| Key results | 18 | Summarise key results with reference to study objectives | 22 | This study revealed that DAO is relatively common among middle-aged and older individuals with T2D and usually presents unique traits related to age and diabetes. More importantly, the presence of DAO appears to be robustly linked to poorer cognitive function, and such a correlation remains significant even after adjusting for key confounding variables, suggesting that DAO, for the first time, has been recognized within diabetic sufferers as a simple, user-friendly, and modifiable marker that may play a pivotal role in potentially preventing and delaying adverse cognitive outcomes. |
| Limitations | 19 | Discuss limitations of the study, taking into account sources of potential bias or imprecision. Discuss both direction and magnitude of any potential bias | 25 | This study has several limitations. First, it is a cross-sectional study, and causality cannot be determined. Fortunately, a preliminary finding emerging from a small-sample study indicated that the novel drug Bimagrumab has the potential to diminish fat mass concurrently with increasing muscle mass[39], and future investigations are needed to determine whether the application of this medication among individuals with diabetes can effectively reduce the risk of cognitive impairment. In addition, this was a single-center study with a small sample size, and further research is needed across a more diverse racial spectrum, within a nondiabetic population, and with an expanded sample size to confirm our conclusions. |
| Interpretation | 20 | Give a cautious overall interpretation of results considering objectives, limitations, multiplicity of analyses, results from similar studies, and other relevant evidence | 25 | In summary, our results indicate that DAO is relatively common among middle-aged and older patients with T2D and clearly displays unique features linked to age and diabetes. Compared with abdominal obesity or dynapenia alone, DAO leads to a synergistically greater risk for cognitive impairment, even after adjusting for diverse confounders. More research is needed to explore whether cognitive decline can be prevented or delayed by improving body composition in subjects with or without diabetes. |
| Generalisability | 21 | Discuss the generalisability (external validity) of the study results | 25 | This was a single-center study with a small sample size, and further research is needed across a more diverse racial spectrum, within a nondiabetic population, and with an expanded sample size to confirm our conclusions. |
| Other information | |  | | |
| Funding | 22 | Give the source of funding and the role of the funders for the present study and, if applicable, for the original study on which the present article is based | 27 | This research did not receive any specific grant from funding agencies in the public, commercial, or not-for-profit sectors. |

*Give information separately for cases and controls in case-control studies and, if applicable, for exposed and unexposed groups in cohort and cross-sectional studies.

**Note:** An Explanation and Elaboration article discusses each checklist item and gives methodological background and published examples of transparent reporting. The STROBE checklist is best used in conjunction with this article (freely available on the Web sites of PLoS Medicine at http://www.plosmedicine.org/, Annals of Internal Medicine at http://www.annals.org/, and Epidemiology at http://www.epidem.com/). Information on the STROBE Initiative is available at www.strobe-statement.org.
