# Supplementary material for: Dynapenic Abdominal Obesity and Cognitive Impairment in Type 2 Diabetic Patients: A Single‐Center Cross‐Sectional Study
Source: Int J Endocrinol. 2026 Mar 12;2026:6060666. doi: 10.1155/ije/6060666 (PMC13098355; doi:10.1155/ije/6060666)
Supplement: Supplementary file 1 — Supporting Information Additional supporting information can be found online in the Supporting Information section. [file IJE-2026-6060666-s001.zip › Supplementary material.docx]

| **Supplementary Table A** | | |  |  |  |  |  |
| --- | --- | --- | --- | --- | --- | --- | --- |
| The appendant demographics and biochemical indicators of participants. | | | | | |  |  |
|  | Category | Overall | Dynapenic  obesity | Nondynapenic obesity | Dynapenic nonobesity | Nondynapenic nonobesity | p |
| n (%) |  | 270 (100.0) | 56 (20.7) | 121 (44.8) | 32 (11.6) | 61 (22.6) |  |
| **Demographics** | |  |  |  |  |  |  |
| Marriage (%) | Married | 212 (78.5) | 39 (69.6) | 98 (81.0) | 24 (75.0) | 51 (83.6) | 0.449 |
|  | Divorce | 23 (8.5) | 5 (8.9) | 10 (8.3) | 3 (9.4) | 5 (8.2) |  |
|  | Widowed | 35 (13.0) | 12 (21.4) | 13 (10.7) | 5 (15.6) | 5 (8.2) |  |
| Living alone (%) | | 34 (12.6) | 8 (14.3) | 14 (11.6) | 5 (15.6) | 7 (11.5) | 0.897 |
| Income (%) | Low | 55 (21.1) | 15 (26.8) | 18 (16.1) | 12 (37.5) | 10 (16.4) | 0.064 |
|  | Middle | 130 (49.8) | 30 (53.6) | 57 (50.9) | 14 (43.8) | 29 (47.5) |  |
|  | High | 76 (29.1) | 11 (19.6) | 37 (33.0) | 6 (18.8) | 22 (36.1) |  |
| Occupation | Farmers | 21 (7.8) | 5 (8.9) | 8 (6.6) | 4 (12.5) | 4 (6.6) | 0.15 |
| (%) | Laborers | 94 (34.8) | 25 (44.6) | 35 (28.9) | 16 (50.0) | 18 (29.5) |  |
|  | Leaders | 79 (29.3) | 12 (21.4) | 38 (31.4) | 6 (18.8) | 23 (37.7) |  |
|  | Professors | 47 (17.4) | 9 (16.1) | 23 (19.0) | 6 (18.8) | 9 (14.8) |  |
|  | others | 29 (10.7) | 5 (8.9) | 17 (14.0) | 0 (0.0) | 7 (11.5) |  |
| **Metabolic indicators** | | |  |  |  |  |  |
| Total cholesterol (mmol/L) | | 4.12 [3.45, 5.05] | 3.86 [3.26, 5.19] | 4.08 [3.36, 4.93] | 4.61 [3.54, 5.77] | 4.39 [3.66, 4.92] | 0.293 |
| Triglyceride (mmol/L) | | 1.30 [0.91, 1.88] | 1.48 [1.00, 1.98] | 1.33 [1.01, 2.02] | 1.00 [0.71, 1.42] | 1.18 [0.77, 1.77] | 0.033 |
| HDL-C (mmol/L) | | 1.13 [0.93, 1.32] | 1.08 [0.93, 1.31] ^c^ | 1.06 [0.91, 1.25] ^c^ | 1.27 [1.12, 1.64] ^a, b^ | 1.17 [0.99, 1.33] | 0.003 |
| LDL-C (mmol/L) | | 2.50 [1.87, 3.27] | 2.29 [1.68, 3.31] | 2.48 [1.85, 3.12] | 2.79 [2.13, 3.75] | 2.59 [2.20, 3.13] | 0.338 |
| Uric acid (umol/L) | | 313.00 [256.50, 385.50] | 326.00 [266.50, 405.50] | 318.00 [254.00, 390.00] | 319.00 [251.00, 393.50] | 301.00 [268.50, 337.50] | 0.321 |
| **Nutritional and inflammatory markers** | | | |  |  |  |  |
| Prealbumin (mg/L) | | 224.82 (49.17) | 215.07 (53.22) | 232.59 (48.31) | 211.10 (44.74) | 225.92 (48.28) | 0.096 |
| Albumin (g/L) | | 42.00 [39.00,44.00] | 41.00 [39.00, 44.00] | 42.00 [40.00, 44.00] | 40.00 [36.75,44.00] | 41.00 [39.00, 44.00] | 0.262 |
| Folic acid (ng/mL) | | 13.50 [9.60, 19.65] | 14.20 [10.65, 18.90] | 13.40 [9.67, 19.85] | 14.05 [10.98,21.98] | 11.50 [8.50, 18.25] | 0.343 |
| Vitamin B12 (pg/mL) | | 364.50 [255.00, 529.75] | 394.00 [287.75, 545.25] | 324.00 [255.00, 505.00] | 348.00 [263.00, 827.50] | 366.00 [262.00, 507.50] | 0.758 |
| 25(OH)D (ng/mL) | | 17.40 [12.90,21.40] | 16.70 [11.90, 20.00] | 17.35 [12.65, 21.83] | 16.95 [13.70,21.45] | 18.30 [13.68, 21.92] | 0.337 |
| IGF-1 (ng/mL) | | 119.00 [89.10, 156.00] | 121.00 [91.05, 159.00] | 119.00 [86.20, 156.00] | 118.00 [102.50, 164.00] | 121.50 [89.55, 149.50] | 0.903 |
|  |  |  |  |  |  |  |  |
| HDL-C, high-density lipoprotein-cholesterol; LDL-C, low-density lipoprotein-cholesterol; 25(OH)D, 25-Hydroxyvitamin D; IGF-1, Insulin-like Growth Factor 1. | | | | | | | |
| ^a^ Significantly different from dynapenic obesity group. | | | | |  |  |  |
| ^b^ Significantly different from nondynapenic obesity group. | | | | |  |  |  |
| ^c^ Significantly different from dynapenic nonobesity group. | | | | |  |  |  |

| **Supplementary Table B** | | | |  |  |  |  |
| --- | --- | --- | --- | --- | --- | --- | --- |
| The appendant diabetes-related features of participants. | | | | | | |  |
|  |  | Overall | Dynapenic  obesity | Nondynapenic obesity | Dynapenic  nonobesity | Nondynapenic nonobesity | p |
| n (%) |  | 270 (100.0) | 56 (20.7) | 121 (44.8) | 32 (11.6) | 61 (22.6) |  |
| **Comorbidities** | |  |  |  |  |  |  |
| CHD (%) |  | 53 (19.8) | 18 (32.1) ^b^ | 15 (12.6) ^a^ | 9 (28.1) | 11 (18.0) | 0.013 |
| Stroke (%) |  | 15 (5.6) | 2 (3.6) | 8 (6.7) | 2 (6.2) | 3 (4.9) | 0.88 |
| **Complications** | |  |  |  |  |  |  |
| Hypoglycemic events (%) | | 89 (33.7) | 22 (39.3) | 34 (29.1) | 11 (34.4) | 22 (37.3) | 0.521 |
| Diabetic nephropathy (%) | | 86 (32.1) | 21 (37.5) | 36 (30.3) ^c^ | 18 (56.2) ^b, d^ | 11 (18.0) ^c^ | 0.002 |
| **Medication** | |  |  |  |  |  |  |
| Metformin (%) | | 196 (73.1) | 37 (66.1) | 94 (79.0) ^c^ | 15 (46.9) ^b, d^ | 50 (82.0) ^c^ | 0.001 |
| Insulin secretagogues (%) | | 13 (4.9) | 4 (7.1) | 5 (4.2) | 1 (3.1) | 3 (4.9) | 0.818 |
| Thiazolidinediones (%) | | 1 (0.4) | 0 (0.0) | 0 (0.0) | 0 (0.0) | 1 (1.6) | 0.557 |
| Insulin (%) |  | 157 (59.2) | 32 (57.1) | 65 (56.0) | 24 (75.0) | 36 (59.0) | 0.274 |
| Statins (%) | | 246 (91.1) | 52 (92.9) | 114 (94.2) | 30 (93.8) | 50 (82.0) | 0.064 |
| RAS blockers (%) | | 116 (43.0) | 28 (50.0) | 56 (46.3) | 15 (46.9) | 17 (27.9) | 0.056 |
|  |  |  |  |  |  |  |  |
| CHD, coronary heart disease; RAS, renin-angiotensin system; | | | | | | | |
| ^a^ Significantly different from dynapenic obesity group. | | | | |  |  |  |
| ^b^ Significantly different from nondynapenic obesity group. | | | | |  |  |  |
| ^c^ Significantly different from dynapenic nonobesity group. | | | | |  |  |  |
| ^d^ Significantly different from nondynapenic nonobesity group. | | | | |  |  |  |
